# A Case of Pulmonary Tumor Thrombotic Microangiopathy Diagnosed by Transbronchial Lung Biopsy and Treated with Chemotherapy and Long-Term Oxygen and Anticoagulation Therapies

**DOI:** 10.1155/2013/259080

**Published:** 2013-08-29

**Authors:** Atsushi Kitamura, Naoki Nishimura, Torahiko Jinta, Rika Suda, Yasuhiko Yamano, Genta Ishikawa, Yutaka Tomishima, Tsuyoshi Hamaoka, Koyu Suzuki, Naohiko Chohnabayashi

**Affiliations:** ^1^Division of Pulmonary Medicine, St. Luke's International Hospital, 9-1 Akashi-cho, Chuo-ku, Tokyo 104-8560, Japan; ^2^Department of Respirology, Graduate School of Medicine, Chiba University, 1-8-1 Inohana, Chuo-ku, Chiba 260-8670, Japan; ^3^Division of Breast Surgical Oncology, St. Luke's International Hospital, 9-1 Akashi-cho, Chuo-ku, Tokyo 104-8560, Japan; ^4^Division of Pathology, St. Luke's International Hospital, 9-1 Akashi-cho, Chuo-ku, Tokyo 104-8560, Japan

## Abstract

A 41-year-old woman, who underwent breast resection for cancer of the right breast and adjuvant chemotherapy 2 years ago, was admitted to our hospital due to shortness of breath upon exertion. High-resolution computed tomography of the chest showed small nodular opacities in the peribronchiolar area in both lungs, as well as mediastinal and hilar lymphadenopathy. A transbronchial lung biopsy revealed breast cancer metastasis and pulmonary tumor thrombotic microangiopathy (PTTM). Treatment of PTTM is rarely reported due to the difficulty of antemortem diagnosis; however, the patient was effectively treated with chemotherapy and oxygen and anticoagulation therapies for 3 months.

## 1. Introduction

Pulmonary tumor thrombotic microangiopathy (PTTM) is a rare complication of malignant diseases that was first reported by von Herbay et al. in 1990 [1]. An antemortem diagnosis of PTTM is difficult, due to the typical development of acute pulmonary hypertension, which causes heart failure, shortness of breath, and death in a few days [2]. PTTM was diagnosed in the present case by a transbronchial lung biopsy (TBLB), despite severe pulmonary hypertension. The patient was effectively treated with chemotherapy and oxygen and anticoagulation therapies.

## 2. Case Report

A 41-year-old Japanese woman, with no history of cigarette smoking or familial cancer, was diagnosed in our hospital 2 years ago with clinical stage 2B (T2N1M0, according to the TNM classification (7th edition) of the International Union Against Cancer) invasive micropapillary carcinoma of the right breast. The immunostaining data for the estrogen receptor (ER), progesterone receptor (PR), and CerbB-2 (HER2) is negative in neoplastic cells. The patient underwent 4 courses of definitive neoadjuvant chemotherapy with cyclophosphamide, epirubicin, and fluorouracil (500 mg/m^2^, 100 mg/m^2^, and 500 mg/m^2^ on day 1, resp.) every 3 weeks and 2 courses of additional chemotherapy with docetaxel (75 mg/m^2^ on day 1) every 3 weeks. Despite chemotherapy treatment, the right breast mass increased in size. Resection of the right breast was performed, and a pathological diagnosis of stage 3B (T3N2M0) breast cancer was made. Following resection, the patient underwent definitive adjuvant chemoradiotherapy with capecitabine (300 mg/body) for 6 months, with concurrent radiation of 50 Gy in 25 fractions. A complete response, confirmed by computed tomography (CT) scanning, was achieved, and the patient was subsequently seen on a regular basis for followup.

**Figure 1:**
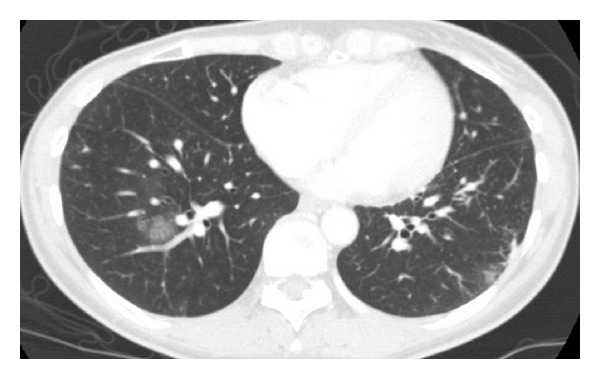
Computer tomography scans of the chest revealed ground glass opacities around the bronchovascular bundles in the lower lobe of the right lung.

**Figure 2:**
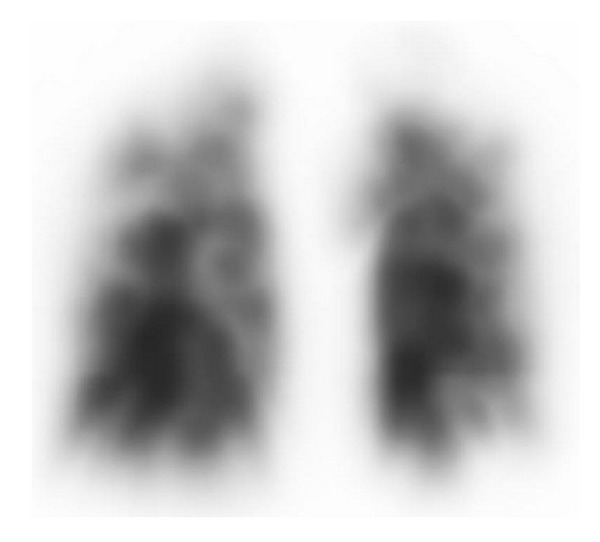
A lung perfusion scan showed multiple wedge-shaped perfusion defects in both lungs.

**Figure 3:**
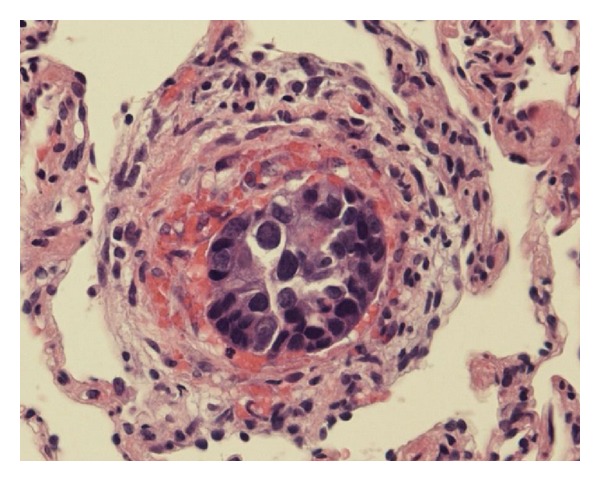
Obstruction of the pulmonary artery and endothelial fibrocystic hyperplasia was determined by transbronchial lung biopsy specimens from the lower lobe of the right lung (hematoxylin and eosin staining, ×40).

One year after definitive treatment, the patient was readmitted to our hospital due to 2 months of shortness of breath upon exertion. No abnormal breath sounds were heard in either of the lung fields. Biochemical examination of the blood revealed significantly elevated lactate dehydrogenase levels (1687 IU/L) (normal < 223 IU/L), moderately elevated aspartate aminotransferase levels (53 IU/L) (normal < 32 IU/L), moderately elevated alanine transaminase levels (62 IU/L) (normal < 38 IU/L), and a moderate abnormality in the coagulation and fibrinolytic system (D-dimer, 12.1 *μ*g/mL) (normal < 1.0 *μ*g/mL). The leukocyte count (3900/*μ*L) was within the normal range, and arterial blood gas analysis showed mild hypoxemia (PaO_2_ 62 mmHg). Although normal results for electrocardiography were seen, echocardiography indicated pulmonary hypertension with tricuspid regurgitation. The estimated gradient pressure was approximately 41 mmHg. CT scans of the chest showed no evidence of pulmonary embolism but revealed ground glass opacities in the lower lobe of the right lung (Figure 1). A perfusion lung scan showed multiple small, wedge-shaped perfusion defects throughout both lungs (Figure 2). PTTM was suspected, and bronchoscopy was performed to determine the pathological diagnosis. TBLB specimens from the right lower lobe determined adenocarcinoma in the pulmonary artery and endothelial fibrocystic hyperplasia in the arterioles (Figure 3). Based upon these histopathological findings, a diagnosis of PTTM was made. Immunohistochemical staining of the tumor specimens showed positive results for cytokeratin AE1/AE3, cytokeratin 5/6, and cytokeratin 14 and a negative result for thyroid transcription factor-1, suggesting breast cancer metastasis.

Following the diagnosis, the patient was treated with the anticoagulant warfarin, 1 course of chemotherapy with irinotecan (80 mg/m^2^ on days 1, 8, and 15), and long-term oxygen therapy. Her shortness of breath upon exertion significantly improved initially; however, her respiratory condition gradually worsened, and follow-up CT scans showed multiple liver and bone metastatic lesions. Echocardiography indicated worsening pulmonary hypertension with tricuspid regurgitation, and the estimated gradient pressure was approximately 100 mmHg. For subsequent treatment, the patient received 1 course of chemotherapy with tegafur, gimeracil, and oteracil potassium (100 mg/body on days 1–21). An initial therapeutic effect was temporarily observed; however, her respiratory condition worsened again, and the patient died of respiratory failure 3 months after the diagnosis of PTTM.

## 3. Discussion

The characteristic histopathological findings of PTTM are tumor cells invading the pulmonary vascular system, fibrocellular and/or fibromuscular intimal proliferation, and luminal stenosis [1, 3]. Patients with PTTM often present with progressive dyspnea and severe pulmonary hypertension [4–9]. It is reported that PTTM presents CT scans of the chest as multiple small nodular opacities or a tree in bud pattern in the peribronchiolar area [10, 11], but these findings are nonspecific. The findings of the CT scans of our patient were also nonspecific. In contrast, we have found that multiple small, wedge-shaped perfusion defects in a perfusion lung scan are characteristic of PTTM and suggest lesions in the blood vessels.

Because PTTM rapidly progresses to death, antemortemdiagnosis is very difficult, and most cases are diagnosed at autopsy [4, 8]. The average days of survival after onset of dyspnea were 16.2 days [12]. However, some antemortem diagnostic methods have been suggested, including video-assisted thoracic surgery [6, 9] and TBLB [7]. In the present case, TBLB was useful for obtaining histopathological specimens and a definitive diagnosis, which allowed treatment to begin immediately. We suggest that, when echocardiography indicates pulmonary hypertension despite slight and nonspecific CT findings, PTTM should be suspected, and random TBLB might be useful for obtaining a definitive diagnosis. Due to the extremely rapid progression of PTTM, few cases have been reported where systemic chemotherapy has improved the prognosis [6, 9, 13]. New drugs for the treatment of pulmonary arterial hypertension, such as endothelin antagonists, prostacyclin analogues, and phosphodiesterase type 5 inhibitors, are controversial for the treatment of PTTM [14]. Because a diagnosis of PTTM using TBLB was made before the disease had progressed to death, the fatal prognosis of the disease that the average days of survival after onset of dyspnea were 16.2 days was ameliorated for 3 months by systemic chemotherapy and long-term oxygen and anticoagulation therapies.

All clinicians should be aware that PTTM causes rapidly worsening respiratory conditions in cancer patients. The early diagnosis by TBLB and use of systemic chemotherapy and long-term oxygen and anticoagulation therapies could improve the fatal prognosis of PTTM.
